# U1 snRNP regulates cancer cell migration and invasion in vitro

**DOI:** 10.1038/s41467-019-13993-7

**Published:** 2020-01-07

**Authors:** Jung-Min Oh, Christopher C. Venters, Chao Di, Anna Maria Pinto, Lili Wan, Ihab Younis, Zhiqiang Cai, Chie Arai, Byung Ran So, Jingqi Duan, Gideon Dreyfuss

**Affiliations:** Howard Hughes Medical Institute, Department of Biochemistry and Biophysics, University of Pennsylvania School of Medicine, Philadelphia, PA 19104-6148 USA

**Keywords:** Cancer, Cell biology, Non-coding RNAs, Transcription

## Abstract

Stimulated cells and cancer cells have widespread shortening of mRNA 3’-untranslated regions (3’UTRs) and switches to shorter mRNA isoforms due to usage of more proximal polyadenylation signals (PASs) in introns and last exons. U1 snRNP (U1), vertebrates’ most abundant non-coding (spliceosomal) small nuclear RNA, silences proximal PASs and its inhibition with antisense morpholino oligonucleotides (U1 AMO) triggers widespread premature transcription termination and mRNA shortening. Here we show that low U1 AMO doses increase cancer cells’ migration and invasion in vitro by up to 500%, whereas U1 over-expression has the opposite effect. In addition to 3’UTR length, numerous transcriptome changes that could contribute to this phenotype are observed, including alternative splicing, and mRNA expression levels of proto-oncogenes and tumor suppressors. These findings reveal an unexpected role for U1 homeostasis (available U1 relative to transcription) in oncogenic and activated cell states, and suggest U1 as a potential target for their modulation.

## Abstract (web summary)

U1 snRNP is a key regulator of mRNA biogenesis through its roles in splicing, and transcription and 3’-end processing. Here the authors show a tumor suppressor-like function of U1 snRNP using in vitro cell migration/invasion assays and transcriptome profiling.

## Introduction

Widespread shortening of messenger RNA (mRNA) 3ʹ-untranslated regions (3ʹUTRs) and switches to short mRNA isoforms is a common feature and contributing factor to cell stimulation, seen in immune cells and neurons, and oncogenicity^1–6^. These shortening events occur due to a shift in usage of more upstream polyadenylation signals (PASs) in the last exon and in introns. In quiescent cells, these PASs are generally silenced by U1 snRNP (U1), vertebrates’ most abundant non-coding small nuclear RNP, which is necessary for production of full-length RNA polymerase II transcripts from protein-coding genes and long non-coding RNAs^7^. This U1 activity, called telescripting, requires RNA base-pairing of U1 snRNA 5ʹ-end, which is also required for U1’s role in 5ʹ splice site (5’ss) recognition. U1 antisense morpholino oligonucleotides (U1 AMO), which inhibits U1:pre-mRNA base-pairing, triggers widespread premature cleavage and polyadenylation (PCPA), as well as inhibits splicing^8,9^. Transfection of a high U1 AMO dose that masks all, or nearly all, of U1 snRNA 5ʹ-end causes drastic PCPA from cryptic PASs frequently found in the 5ʹ-side of the intron in pre-mRNAs of thousands of genes^8,9^, especially in long introns of large genes (>39 kb). In contrast, small genes (<6.8 kb) are generally PCPA-resistant and many of them are upregulated in this environment. Importantly, these small genes are enriched in functions related to cell survival and acute cell stress response^7^. The drastic PCPA from high U1 AMO in many genes obscures other effects that are more readily detected with low U1 AMO doses. These changes include 3ʹUTR shortening (shifts to usage of more proximal PASs in tandem PASs) and the production of shorter mRNA isoforms from PAS usage in introns^9^. This revealed that low U1 AMO recapitulates known shifts to shorter mRNA isoforms that occurs in stimulated neuronal cells. We have shown previously that the stimulation-induced rapid transcription upregulation creates transient telescripting deficit because U1 levels cannot rise in step with the transcription surge from immediate early genes^9^. U1 synthesis is a slower process involving nuclear export of pre-snRNAs, SMN complex-mediated snRNP assembly in the cytoplasm, and re-import to the nucleus^10^. Consequently, some pre-mRNAs transcribed in this time window (~2–6 h post stimulation) are processed to shorter mRNA isoforms due to PCPA in introns. For example, pre-mRNA processing of *homer-1*, which encodes a synaptogenesis scaffold protein, shifted from full-length mRNA to a shorter mRNA isoform that encodes antagonistic activity due to PCPA in an intron^11^. Importantly, low U1 AMO recapitulated the same isoform switching^9^.

Here, we investigated if low U1 AMO and U1 over-expression could also modulate cell phenotype. Our studies uncover a role for U1 in regulating cancer cells’ phenotype, and identify widespread and diverse transcriptome changes resulting from modulating U1 availability. These changes are consistent with U1’s central role in splicing and telescripting, and include many with known functions in cancer.

## Results

### U1 level changes modulate hallmark phenotypes of cancer cells in vitro

**Fig. 1:**
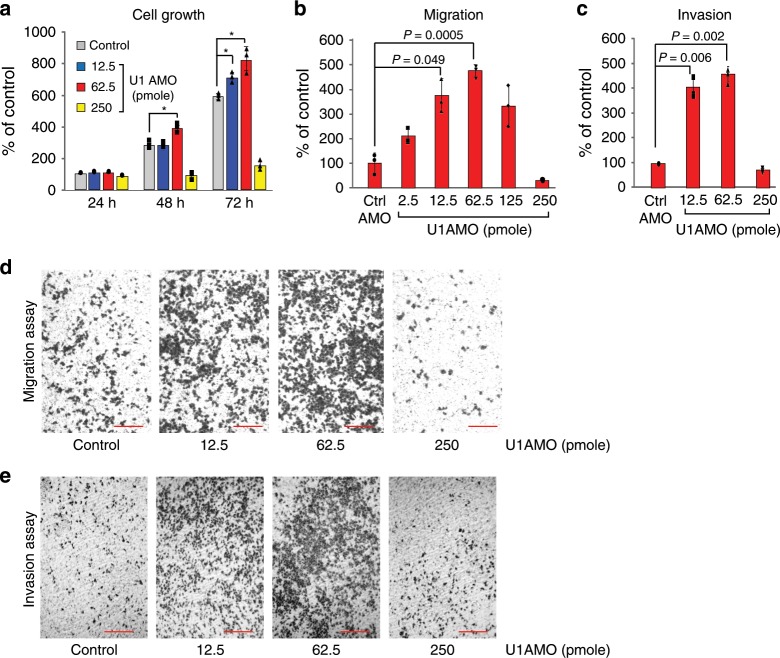
Low U1 AMO dose-dependently enhances migration and invasion of HeLa cells in vitro. **a** HeLa cells were transfected with the indicated amounts of U1 or control AMOs. Cell proliferation was determined from cell numbers using the Cell Titer-Glo Luminescent Cell Viability Assay at the times indicated. Data are represented as mean ±  standard deviation (SD) (*n* = 3, independent cell culture). *P*-value was calculated with two-tailed Student’s *t*-test. The asterisk indicates *P* < 0.05. **b**, **c** Migrating and invading cells were measured 24 h post-transfection. 1.5 × 10^5^ and 2.5 × 10^5^ cells were cultured for the migration and invasion assays, respectively. Cells migrated and invaded from an upper chamber to a bottom chamber over 24 h and were counted using the cell staining solution Cytoselect 24-well cell migration assay kit (Cell Biolabs). The number of migrated and invaded cells were compared with each control. Data are represented as mean ± SD (*n* = 3, independent cell culture). *P*-value was calculated with two-tailed Student’s *t*-test. **d**, **e** Migrating and invading cells were stained 24 h after transfection and visualized by phase-contrast microscopy (10x magnification). The number of migrated cells were 111, 344, 472, and 26 in control, 2.5, 62.5, and 250 U1 AMO (pmole), respectively. The number of invaded cells were 124, 472, 532, and 88 in control, 2.5, 62.5, and 250 U1 AMO (pmole), respectively. Scale bar = 250 µm. Source data are provided as a Source Data file.

We used standard in vitro assays to determine if moderate U1 inhibition has an effect on proliferation, migration, or invasion of cancer cells, which serve as quantitative measures of oncogenic phenotype. Various low U1 AMO doses (2.5–250 pmole) or control, non-targeting AMO (cAMO) were transfected into HeLa cells, a cervical carcinoma cell line (Fig. 1). These U1 AMO doses masked ~15–30% of U1 snRNA 5ʹ-ends, making it inaccessible for base-pairing corresponding to the U1 AMO dose^8,9^. As shown in Fig. 1a, 62.5 pmole U1 AMO moderately increased (38%) cell proliferation after 48–72 h. Higher U1 AMO doses (≥250 pmole) were toxic and reduced overall cell numbers. Remarkably, low U1 AMO dose-dependently enhanced cell migration and invasion by up to ~500% in 24 h with peak activity at 62.5 pmole (Fig. 1b–e). The increased migration and invasion reflect true enhancements that could not be accounted for by the comparatively small increase in cell number (Fig. 1a). U2 AMO, which interferes with U2 snRNP’s function in splicing^12^, did not enhance any of these phenotypes over the entire dose range, indicating that the specificity of the U1 AMO effects (Supplementary Fig. 1).

**Fig. 2:**
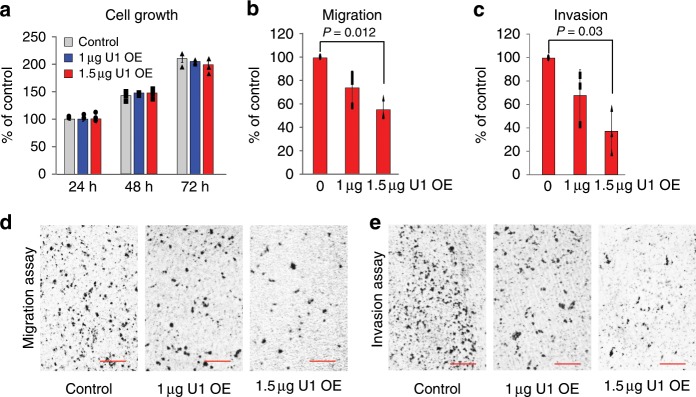
U1 over-expression decreases cell migration and invasion of HeLa cells in vitro. HeLa cells were transfected with control empty vector or U1 expression plasmids. **a** Cell growth determined in 24 h intervals. The data are represented as mean ± SD (*n* = 3, independent cell culture). **b**, **c** Cell migration and invasion were measured 24 h after transfection. The bars indicate mean ± SD (*n* = 3, independent cell culture). *P*-value was calculated with two-tailed Student’s *t*-test. **d**, **e** Cell migration and invasion were determined 24 h post-transfection and imaged by phase-contrast microscopy (10x magnification). Scale bar = 250 µm. Source data are provided as a Source Data file.

To further examine the effects of U1 on cell phenotype, we over-expressed U1 (U1 OE) from a plasmid carrying U1 snRNA gene with its native promoter and termination elements. This achieved 20% and 40% U1 level increases compared to empty plasmid by transfecting 1 µg and 1.5 µg of this plasmid, respectively (Supplementary Fig. 2). The U1 OE significantly and dose-dependently attenuated cell migration (25–50%) and cell invasion (25–65%) after 24 h (Fig. 2). U1 OE dose-dependently increased the amount of U1 snRNP, determined by the amount of U1 snRNA in anti-Sm immunoprecipitations^9^.

Similar experiments on other cancer cells, including human lung adenocarcinoma (A549) and breast adenocarcinomas (MCF-7 and MDA-MB-231), demonstrated the generality of the U1-related effects on phenotype. U1 AMO enhanced migration of A549, MCF-7, and MDA-MB-231 by 58–72% and increased invasion of A549 and MDA-MB-231 by 53–64% (Supplementary Table 1). Conversely, U1 OE attenuated the migration and invasion of these cell lines by ~50% compared to the control levels (Supplementary Table 1).

### U1 level changes cause numerous and diverse transcriptome changes

We used high-throughput RNA sequencing (RNA-seq) to determine transcriptome effects resulting from U1 level modulation. To enhance detection of nascent RNAs, as U1 functions co-transcriptionally^7^, we metabolically labeled RNAs with 4-thiouridine (4-shU) for 2 h (at 6–8 h post-transfection with AMOs and 22–24 h of U1 OE), and sequenced the thiol-selected RNAs. This protocol also readily detects near steady-state mRNAs. Reads were mapped to the human genome (UCSC, hg38) and filtered for unique alignments. The statistics of the RNA-seq datasets, normalized for sequencing depth as reads per million (RPM), are shown in Supplementary Table 2. Over 9100 genes expressed to RPKM ≥ 1 in the control samples for U1 AMO and U1 OE, and were included in further analysis. This revealed numerous and diverse transcriptome changes caused by U1 AMO, including in mRNA expression, 3ʹUTR length, and alternative splicing affecting thousands of genes. The number of genes affected by each type of change are listed in Supplementary Table 3. Frequently, more than one type of change was detected in transcripts of the same gene (18–47%; Supplementary Fig. 3). Generally, the number of events for each type of change increased with U1 AMO dose and there was extensive overlap (31–97%) between events detected at the two doses tested (Supplementary Table 3). To maintain a high confidence in the transcriptome changes that were analyzed, we did all further analyses and discussion on events that were detected in both doses of U1 AMO or U1 OE, respectively.

**Fig. 3:**
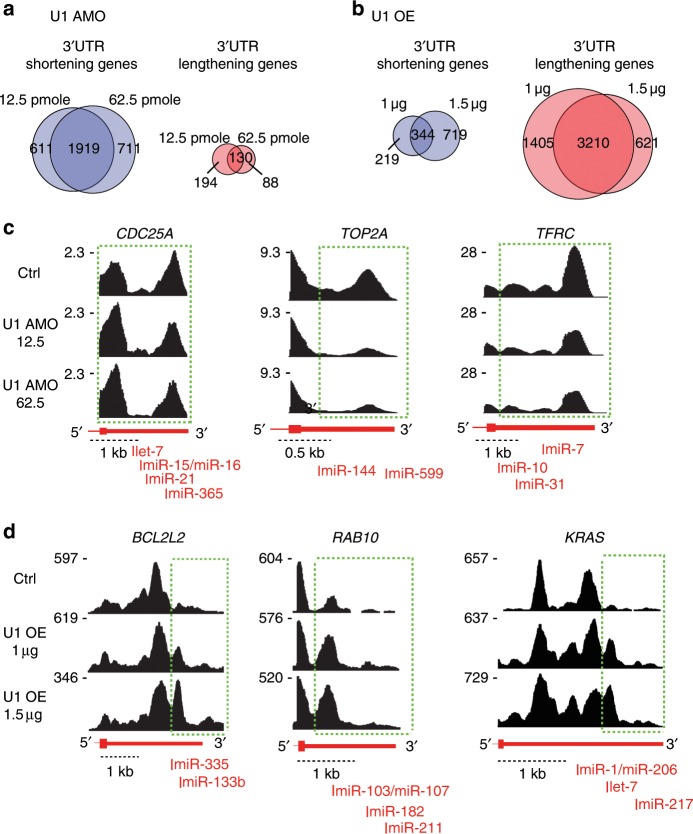
Examples of genes with 3’UTR shortening by U1 AMO and genes with 3’UTR lengthening from U1 over-expression. Venn diagrams of LECDS-identified genes affected by 3ʹUTR shortening versus 3ʹUTR lengthening in U1 AMO 12.5 and 62.5 pmole (**a**) and U1 OE 1 and 1.5 µg (**b**) samples. **c** Cells transfected with control or U1 AMO (12.5 and 62.5 pmole, 8 h) were labeled with 4-thiouridine to select and sequence nascent transcripts. RNA-seq maps of representative genes from two biological experiments are shown. Green dotted boxes indicate the affected regions where shortening in the 3ʹUTR occurs. **d** Cells transfected with control or U1 expression plasmids (1 µg and 1.5 µg, 24 h) were labeled with 4-thiouridine to select nascent transcripts. Green dotted boxes indicate the regions where there was a switch towards longer 3ʹUTRs. miRNA target sites that are involved in cancer and expressed in HeLa are shown.

Confirming earlier observations from datasets with lower resolution and sequencing depth^9^, low U1 AMO elicited widespread 3ʹUTR shortening readily detected in genome browser views, for example, *TOP2A*, *TFRC*, and *CDC25A* (Fig. 3). A common approach to identify changes in locations of 3ʹ-poly(A)s is to use oligo(dT)-primed RNA-seq^13^. However, it does not provide adequate information on overall transcriptome changes due to inherent 3ʹ bias. An alternative method, DaPars, uses a regression model to deduce alternative PAS usage among multiple tandem 3ʹUTR PASs from standard RNA-seq^14^. Frequently, 3ʹUTRs have multiple tandem PASs, resulting in complex mixtures of mRNAs with various 3ʹUTR lengths that make it difficult to resolve by DaPars. We reasoned that a shift in usage to proximal 3ʹUTR PASs would decrease the overall amount of transcription in the 3ʹUTR, while a shift to more distal PASs will increase it. As changes in 3ʹUTR amount could result from mRNA expression level changes alone, we calculated the ratio of RNA-seq reads in the 3ʹUTR to the reads in the portion of the CDS in the same last exon (heretofore LECDS). This normalizes 3ʹUTR signal to mRNA expression. A decrease or increase in LECDS in a sample compared to control suggests 3ʹUTR shortening or lengthening, respectively.

LECDS-identified 3ʹUTR shortening in 1919 genes in both 12.5 and 62.5 U1 AMO (*p* ≤ 0.01), and only a small fraction (130 genes) had more reads in 3ʹUTRs (Fig. 3a). In contrast, U1 OE caused a 3ʹ increase in reads (longer 3ʹUTRs) in 3210 mRNAs, while it shortened 3ʹUTRs in only 344 genes (Fig. 3b). The large overlap of the lengthened (~84%) and shortened genes (~76%) in both U1 OE (1 µg and 1.5 µg) and U1 AMO samples (U1 AMO 12.5 and 62.5), respectively, showed a strong validation of the RNA-seq data from separate biological experiments. Although LECDS is a much simpler calculation and lacks the ability to define the location of the PAS used, it nevertheless called 3ʹUTR shortening in 79% of the mRNAs identified as such by DaPars (Supplementary Fig. 4). Further confirmation of 3ʹUTR shortening and lengthening is shown for select examples in Fig. 3c, d, and 3ʹRACE shows that they result from APA (Supplementary Fig. 5). Notably, the fraction of genes that had 3ʹUTR length changes corresponded to the U1 AMO or OE dose, and U1 OE caused transcription in some genes to extend farther downstream from canonical gene ends. Thus, U1 has a role in regulation of both proximal and distal PASs.

**Fig. 4:**
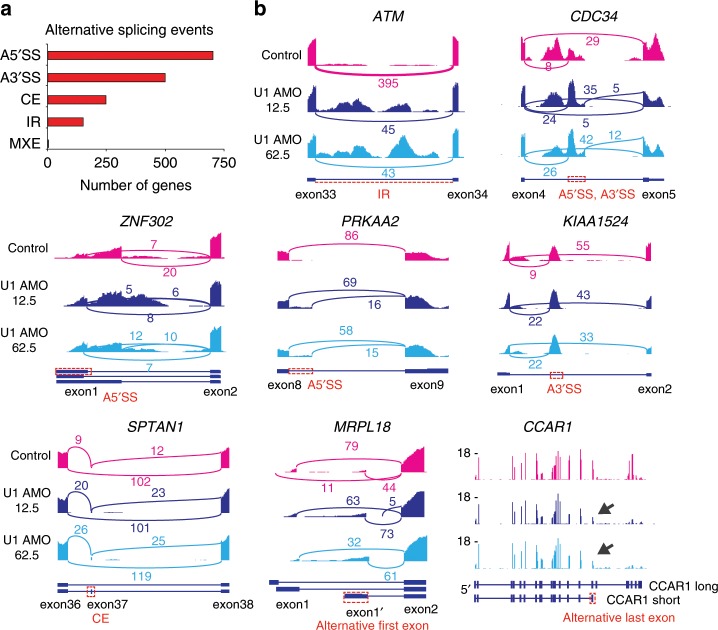
Low U1 AMO causes widespread alternative splicing changes in HeLa cells. **a** Summary of alternative splicing events as identified with JUM analysis. Each alternative splicing event happened in both low U1 AMO 12.5 and 62.5 pmole in HeLa cells. (A5’SS: alternative 5ʹ splice site; A3’SS: alternative 3ʹ splice site; CE: cassette exon; IR: intron retention; MXE: mutually exclusive exons) **b** Sashimi plots and genome browser view of each alternative splicing change in the low U1 AMO 12.5 and 62.5 pmole.

**Fig. 5:**
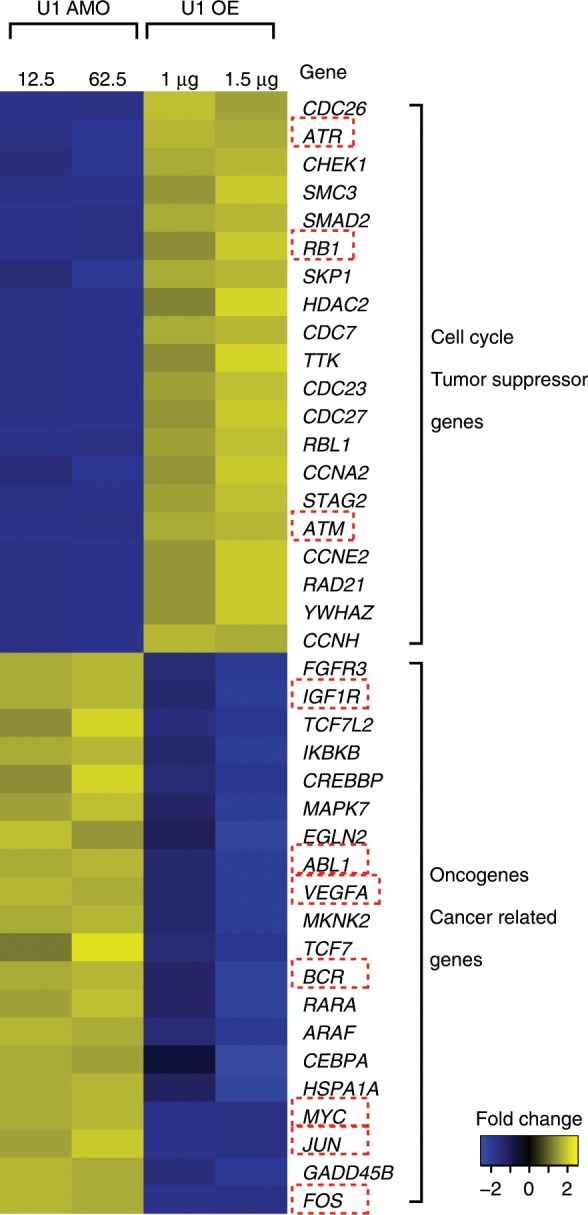
U1 AMO upregulated oncogenes and downregulates tumor suppressor genes and U1 over-expression antagonizes the effect in HeLa cells. Heat map showing genes related to cell cycle and tumor suppressor function are downregulated in U1 AMO and upregulated in U1 OE. Oncogene and cancer related genes are upregulated in U1 AMO and downregulated in U1 OE (1.5-fold change, *P*-value < 0.01 calculated by GFold^55^). Source data are provided as a Source Data file.

As expected for U1’s role in splicing, U1 AMO caused alternative splicing (AS) changes in ~700 genes, including A5’SS and A3’SS, cassette exon (CE) and intron retention (IR) events (Supplementary Table 3 and Fig. 4)^15^. Examples of AS and confirmation of select cases by RT-PCR are shown in Fig. 4b and Supplementary Fig. 6. Many of the observed AS changes could contribute to the oncogenic phenotype. For example, ataxia-telangiectasia, a cancer predisposition disorder caused by mutations in the ATM gene^16^, has two introns retained (intron 1 and 33) and is downregulated in U1 AMO compared to cAMO (Figs. 4b and 5). A similar number of splicing changes is observed with knockdowns of other splicing factors^15,17,18^.

Gene ontology analysis (GO term) revealed expression level changes induced by U1 level modulation in multiple oncogenes, tumor suppressors and cell cycle related genes. For example, *ATR*, *RB1*, *ATM*, and several *CDC* (Cell division cycle) genes were downregulated in U1 AMO, but upregulated in U1 OE. On the other hand, oncogene and cancer related genes, such as *MYC*, *FOS*, *JUN*, and growth promoting genes were upregulated in U1 AMO, but downregulated in U1 OE (Fig. 5 and Supplementary Fig. 7). Downregulation could be explained by PCPA in an intron, which is frequently difficult to detect without exosome inhibition as they are rapidly eliminated^19–21^. Alternatively, downregulation could be caused by inhibition of transcription initiation, as a secondary effect to downregulation or mis-splicing of transcription factors. Upregulation could be caused by PCPA in long genes, which could decrease competition for transcription and splicing factors for non-PCPAed genes, as well as in response to cell stress resulting from PCPA in other genes^7,22^. Nevertheless, concomitant upregulation of oncogenes and downregulation of tumor suppressor genes likely contribute to the phenotypic changes in the wake of U1 AMO. Among genes affected by U1 AMO are splicing factors, which have been linked to myelodysplastic syndromes, chronic lymphocytic leukemia and other cancers (Supplementary Fig. 8)^17,23–25^.

### U1 level changes alters expression of cancer genes

To explore the potential role of U1 dependent 3ʹUTR length changes on the oncogenic phenotype we observed, we interrogated our gene set against cancer gene databases [Sanger^26^ and UCSF (waldman.ucsf.edu/GENES/completechroms.html)]. These archives include oncogenes induced through various mechanisms, including mutation, chromosomal translocation, or loss of miRNA repression, and the analysis revealed that a large number of oncogenes incurred 3ʹUTR length changes. The oncogenes affected at each U1 level, 204 in total, are listed in Supplementary Table 4 and include cell cycle regulation (*CDC25A*, *CCNB1*), apoptosis (*BCL6*, *BRCA1*), cell migration (*FGFR1*, *FYN*), extracellular matrix remodeling (*TIMP2*), signaling (*EGFR*), transcription (*WNT5A*), metastasis and tumor progression (*EWSR1*, *APC*, *BRAF*).

There are numerous examples of cancers resulting from oncogene upregulation due to loss of miRNA repression, either because the relevant miRNA is downregulated or its target in the 3ʹUTR has been removed^27–30^. U1 level changes recapitulated the same miRNA 3ʹUTR target elimination or restoration in many genes (Fig. 3c, d and Supplementary Fig. 5). For CDC25A, an essential phosphatase for the G1-S transition, 3ʹUTR shortening eliminates several miRNA-binding sites, including let-7, miR-15, and miR-21 (Fig. 3c). An increase in CDC25A protein due to alleviation of miRNA-mediated repression exacerbates hepatic cyst formation and colon cancer^31–33^. 3ʹUTR shortening in RAS oncogene family member, *RAB10*, which removes target sites for miRs-103/107, is found in numerous cancer cell lines and results in a dramatic increase of this protein^4^. As shown in Fig. 3d, 3ʹUTR of *RAB10* is already shortened in HeLa cells, and U1 OE reverses this shortening and restores the corresponding miRNA-binding sites. Similarly, U1 OE lengthened 3ʹUTR of *KRAS* to include a let-7 binding site (Fig. 3d). This region contains a polymorphism that has been shown to impair let-7 binding, and is prognostic for breast cancer aggressiveness^34^. U1 AMO also did not change the associated miRNA expression (Supplementary Fig. 9). In addition to loss or gain of miRNA-binding sites, 3’UTR length or amount changes can affect other mRNA regulating elements, such as AU-rich elements (AREs), with roles in cancer phenotype. For example, U1 OE-induced 3ʹUTR lengthening in *c-Fos*, a gene controlling cell proliferation, differentiation, survival and tumorigenesis^35,36^, restores an ARE and strongly decreases mRNA level (>2-fold, Fig. 5).

## Discussion

The numerous transcriptome changes resulting from moderate perturbation of U1 homeostasis, namely, available U1 relative to the amount of transcription, fall into clear categories that can be explained by U1’s known functions in telescripting and splicing. Many of the changes are known to be oncogenic on their own, in humans and mice. For example, U1 AMO recapitulates activation of proto-oncogenes, including 3ʹUTR shortening (e.g., *EGFR*) or upregulation (e.g., *MYC*), and downregulation or ORF-disruptive splicing changes in tumor suppressors (e.g., *ATM*). While it is possible that a change in one or a few of the affected genes is particularly important, our study strongly suggests that the cumulative effect of multiple transcriptome changes that affect a fraction of transcripts in many relevant genes drives the phenotype changes. Our findings demonstrate that U1 homeostasis plays an important role in maintaining normal gene expression balance, and prevent the transcriptome from defaulting to a state in which multiple oncogenic drivers are activated and counter-acting tumor-suppressors are diminished. In that sense, U1’s activity is tumor suppressor-like.

Several other factors that can regulate 3′UTR length have been described, particularly components of the cleavage and polyadenylation machinery (CPA)^13,37–41^. Knockdown of CPSF6/CFIm68, CPSF5/CFIm25, and PABPN1 cause widespread 3ʹUTR shortening^40,42,43^. Furthermore, CPSF5 and CPSF6 are downregulated in cancers and their knockdowns in cell lines enhances oncogenicity^14,40,44,45^. It is possible that these CPA factors (CPAFs) work in concert with U1, as recent evidence for a complex of U1 with CPAFs suggests^22^. 3ʹ UTR shortening analysis of the RNA-seq of CPSF5 knockdown in HeLa cells by LECDS-identified 1210 genes (63% overlap with U1 AMO) that are also shortened in U1 AMO (Supplementary Fig. 10), suggesting potentially extensive commonality of targets. A recent study described that U1 over-expression in PC-12 rat neuronal cell line upregulated cancer related genes, including MYC and FOS^46^. While these results appear to be inconsistent with our observations, they may be explained by the different cell types used in these studies and by different U1 levels relative to transcription.

Mutations in several splicing factors, particularly U2-associated factors and SR proteins, have been shown to drive many cancers^23,24^. However, the potential role of U1 in cancer has not been tested previously. It is intriguing that a major U1 gene cluster in humans at chr1p36.13 flanks a breakpoint linked to many cancers^47^. The potential effect of these genomic abnormalities on U1 levels remains to be determined, as well as U1’s potential role in tumors. Mounting evidence indicate that U1 AMO and U1 over-expression mimic biological processes^7,9,20,48–50^. Thus, these experimental tools for dose-dependent modulation of U1 should facilitate studies on cancer and stimulated cells behavior, and suggest U1 as a potential clinical target for a wide range of disorders.

## Methods

### Cell culture and treatment

Cells (HeLa, A549, MCF-7 and MDA-MB-231 obtained from ATCC) were maintained in Dulbecco's Modified Eagle Medium supplemented with 10% fetal bovine serum (FBS), 10 units ml^−1^ penicillin and 10 μg ml^−1^ streptomycin at 37 °C, and 5% CO_2_. All cell lines were tested for mycoplasma contamination and were not authenticated. U1, U2 antisense, and control morpholino oligonucleotide sequences were 5ʹ-GGTATCTCCCCTGCCAGGTAAGTAT-3ʹ, 5ʹ-CCTCTTACCTCAGTTACAATTTATA-3ʹ and 5ʹ-TGATAAGAACAGATACTACACTTGA-3ʹ, respectively^8^. Oligonucleotides were transfected into 1 × 10^6^ cells using a Neon transfection system (Invitrogen) according to the manufacturer’s instruction to achieve the desired final doses indicated in the text and figures.

### Proliferation assay

The Cell Titer-Glo Luminescent Cell Viability Assay (Promega) was used according to the manufacturer’s instructions to measure cell proliferation. The cells were transfected with AMOs and seeded in triplicate in 96-well plates at a density of 1 × 10^4^ cells per well. Cells were incubated in media containing 1% FBS for 3 days and proliferation was measured every 24 h.

### Migration and invasion assays

For migration studies, a standard assay was used to determine the number of cells that traversed a porous polycarbonate membrane in response to a chemo-attractant (higher serum concentration)^51,52^ using the Cytoselect 24-well cell migration assay (Cell Biolabs). Invasion was measured using BD BioCoat Matrigel invasion chambers (BD Bioscience). In both assays, cells were transfected with AMOs, and 1.5 × 10^5^ and 2.5 × 10^5^ cells per well were seeded in an upper chamber in serum free media for the migration and invasion assay, respectively. The lower chamber was filled with media containing 10% FBS. After 24 h, cells passing through polycarbonate membrane were stained and counted according to the manufacturer’s instructions.

### Metabolic RNA labeling, isolation, and RNA-seq

4-thiouridine (250 μM) was added to cells between 6–8 h after U1 AMOs transfection. Total RNA was extracted with Trizol (Invitrogen) and poly(A) RNA purified on Oligotex beads (Qiagen). Free thiols on poly(A) mRNA were reacted with 0.2 mg ml^−1^ biotin-HPDP for 2 h to label RNA that incorporated 4-thiouridine. RNA was then purified on M-280 streptavidin Dynabeads (Invitrogen), cDNA was synthesized using Ovation RNA-Seq System V2 (NuGEN) and libraries for Illumina sequencing were constructed using Encore NGS Library System (NuGEN) according to the manufacturer’s instructions.

### Mapping RNA-seq reads

RNA-seq reads were aligned to reference genome UCSC/hg38 using STAR^53^ version 2.6.0b with default settings. Reads per exon were grouped, from which RPKM (Read Per Kilobase of exon model per Million mapped reads)^54^ values were calculated. Only genes with RPKM ≥ 1 were included in further analyses.

### Differential gene expression, alternative splicing, and miRNA analyses

The GFold algorithm^55^ was used to calculate differential expression using the default parameters for detecting reliable expression change on all exons of the longest full-length isoform excluding the 3ʹUTR. A 1.5-fold change was set with a *P*-value < 0.01 to identify genes with significant expression change. The Junction Usage Model (JUM v2.0.2)^15^ analysis was performed to identify differentially spliced isoforms in experimental samples compared to controls and quantify their expression levels by computing the ∆PSI (difference of Percent Spliced Isoform). Significant differential alternative splicing events were detected by performing a *Χ*^2^ likelihood-ratio test followed by Benjamini–Hochberg (BH) multiple testing with an adjusted *P*-value ≤ 0.01. For stringent inclusion, we also restricted ∆PSI > = 10%. TargetScan miRNA regulatory sites were downloaded from UCSC Genome browser (http://genome.ucsc.edu/). The coordinate of each predicted miRNA site was compared to those genes for which the 3ʹUTR showed changes in length. For miRNA microarray, HeLa cells were transfected with control or 62.5 pmole U1 AMO for 8 h. Total RNA was extracted using miRNeasy kit (Qiagen) according to manufacturer recommendations. GeneChip miRNA 3.0 array (Affymetrix) was used to determine the miRNA expression and experiments and analysis were performed by Molecular Profiling Facility at University of Pennsylvania.

### LECDS calculations

We used both the 3ʹUTR and coding sequence of the terminal exon to determine the change in reads in 3ʹUTR of the longest (full-length) isoform. As 3ʹUTR reads can change with expression level changes, such as transcription up- or downregulation, 3ʹUTR signals were compared to that from the last exon’s coding sequence (LECDS). If the 3ʹUTR reads are significantly decreased or increased relative to those from the LECDS, the 3ʹUTR is called as shortening or lengthening, respectively. The significance of this read change was detected using a Fisher’s Exact test followed by Benjamini–Hochberg (BH) multiple testing with an adjusted *P*-value ≤ 0.01. This provides an accurate read-out of the net change in 3ʹUTR expression in a gene’s transcript and can be used based on total RNA-seq without specialized poly(A) mapping.

### Reporting summary

Further information on research design is available in the Nature Research Reporting Summary linked to this article.

## Supplementary information

Supplementary Information

Reporting Summary

## Data Availability

All sequencing data described are available on GEO under the accession number GSE140543. The source data underlying Figs. 1a–c, 2a–c, 5 and Supplementary Figs. 1a, b, 2, 5, 6, 7b, d are provided as a Source Data file. All data are available from the corresponding author upon reasonable request.

## Source data

Source data

## Code availability

Code for the analyses described in this study is available from the corresponding author upon request.
