# Supplementary material for: U1 snRNP regulates cancer cell migration and invasion in vitro
Source: Nat Commun. 2020 Jan 7;11:1. doi: 10.1038/s41467-019-13993-7 (PMC6946686; doi:10.1038/s41467-019-13993-7)
Supplement: Supplementary file 1 — Supplementary Information [file 41467_2019_13993_MOESM1_ESM.pdf]

Supplementary information

U1 snRNP regulates cancer cell migration and invasion in vitro

Oh et al.

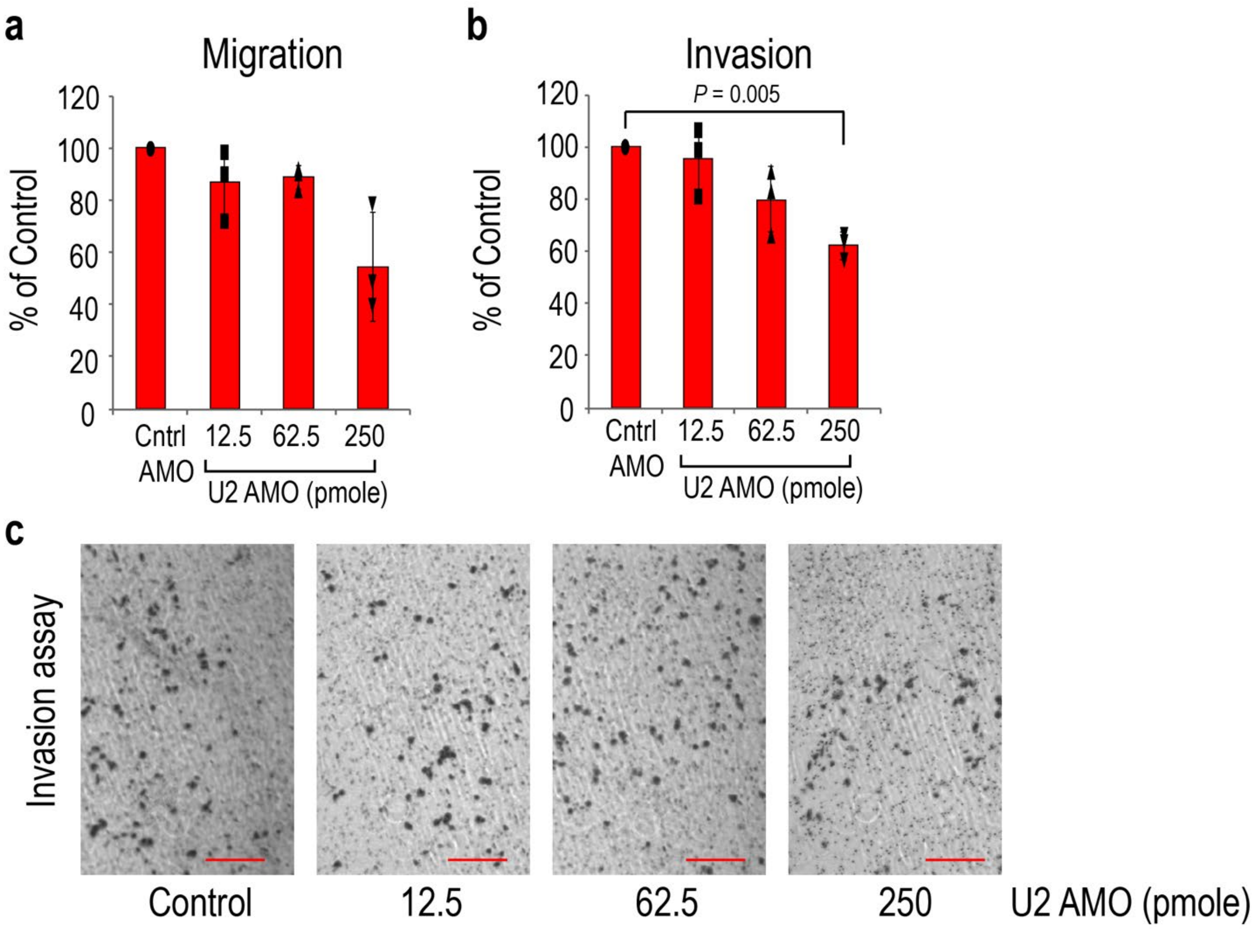

Supplementary Fig. 1. U2 AMO does not enhance migration and invasion of HeLa cells *in vitro*. **a** Migration assay (**b**) invasion assay, and (**c**) phase-contrast imaging 24 hours following transfection with control or U2 AMO (12.5-250 pmole) were performed as described in Fig 1. Data are represented as mean ± SD (n=3, independent cell culture). *P*-value was calculated with two-tailed Student's t-test. U2 AMO at 250 pmole decreased cell numbers. Scale bar = 250μm. Source data are provided as a Source Data file.

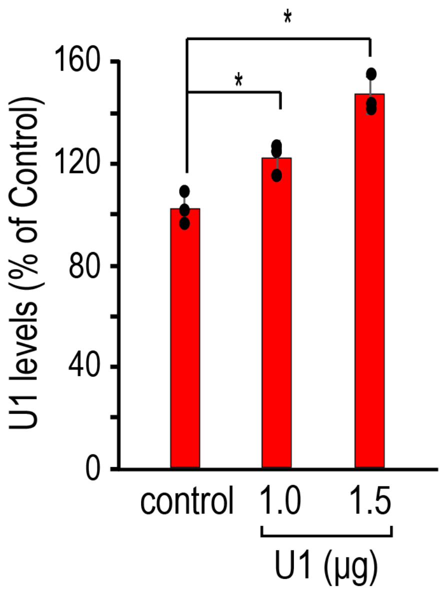

Supplementary Fig. 2. Expression of exogenous U1 snRNA. HeLa cells were transfected with increasing concentrations of a plasmid expressing U1 snRNA under the control of its native promoter for 24 hours. Total U1 levels in total RNA were then determined by RT-qPCR. The RNA input was normalized to 5S rRNA. Data are represented as mean ± SD (n=3, independent cell culture). *P*-value was calculated with two-tailed Student's t-test. The asterisk indicates $p < 0.05$. Quantitation of U1 snRNA assembled into snRNPs immunopurified with anti-Sm antibodies, showed the same U1 increase[1]. Source data are provided as a Source Data file.

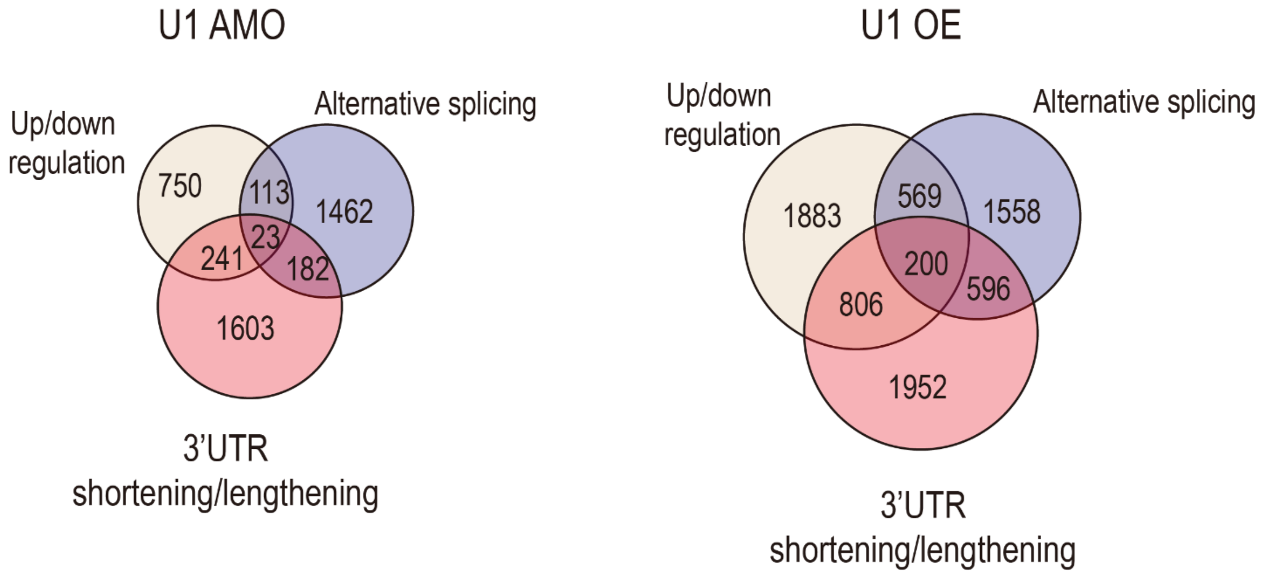

Supplementary Fig. 3. Transcriptome changes induced by U1 AMO and U1 over-expression. Venn diagrams showed the number of overlapped genes from 3 different transcriptome changes: up- and down-regulation, 3'UTR length change, and AS in U1 AMO and U1 OE samples.

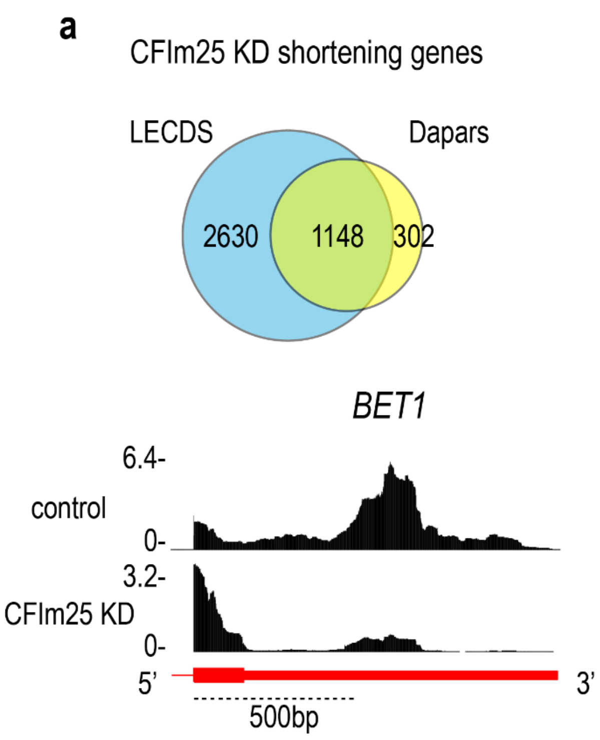

Supplementary Fig. 4. Validation of LECDS calculation. a Significant overlap (79%) between 3'UTR shortening genes captured by LECDS and DaPars[2] calculations. The higher number of 3'UTR shortening events in LECDS (2,630) compared to DaPars may be due to changes in usage at multiple PASs in the same 3'UTR, rather than a strong increase in usage of a single proximal alternative PAS, which is required for DaPars. **b** Genome browser examples show the 3'UTR shortening genes captured by LECDS but not by DaPars.

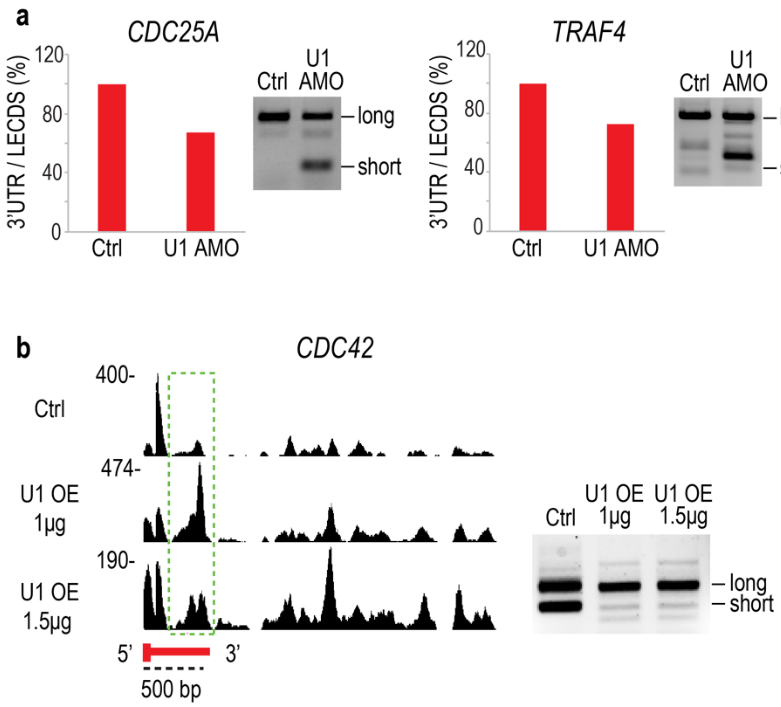

Supplementary Fig. 5. Validation of 3' UTR length changes after U1 AMO and U1 over-expression. Cells were transfected with control or U1 AMO 62.5 pmole for 8 hours, or U1 expression plasmid for 24 hours. Total RNA was extracted using Trizol followed by selection of nascent transcripts. **a** Histogram shows a percentage of 3'UTR / LECDS in each shortening gene that was selected by LECDS, and which was confirmed by 3' RACE. **b** Green dotted boxes in *CDC42*'s 3'UTR indicate the region of the 3'UTRs that was affected by U1 OE, and which was confirmed by 3' RACE. Source data are provided as a Source Data file.

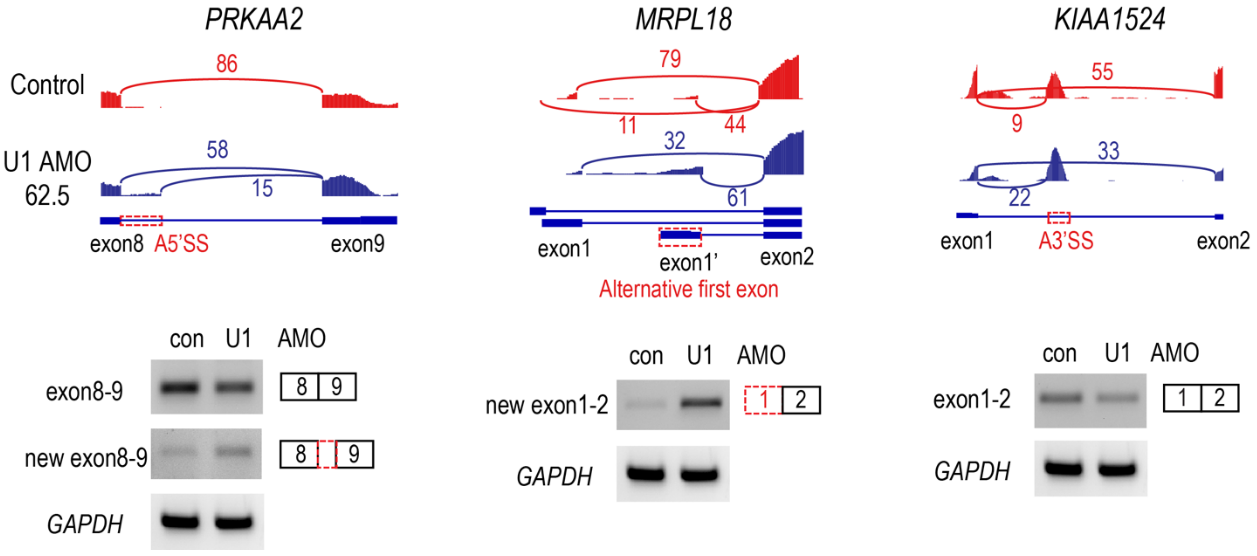

Supplementary Fig. 6. RT-PCR validation of U1 AMO induces alternative splicing changes. HeLa cells transfected with control or U1 AMO 62.5 pmole were incubated with 4-shU in last 2 hours at 6 hours post transfection. 4-shU labeled RNA were purified and used for RT-PCR to confirm the alternative splicing change in each example gene. Source data are provided as a Source Data file.

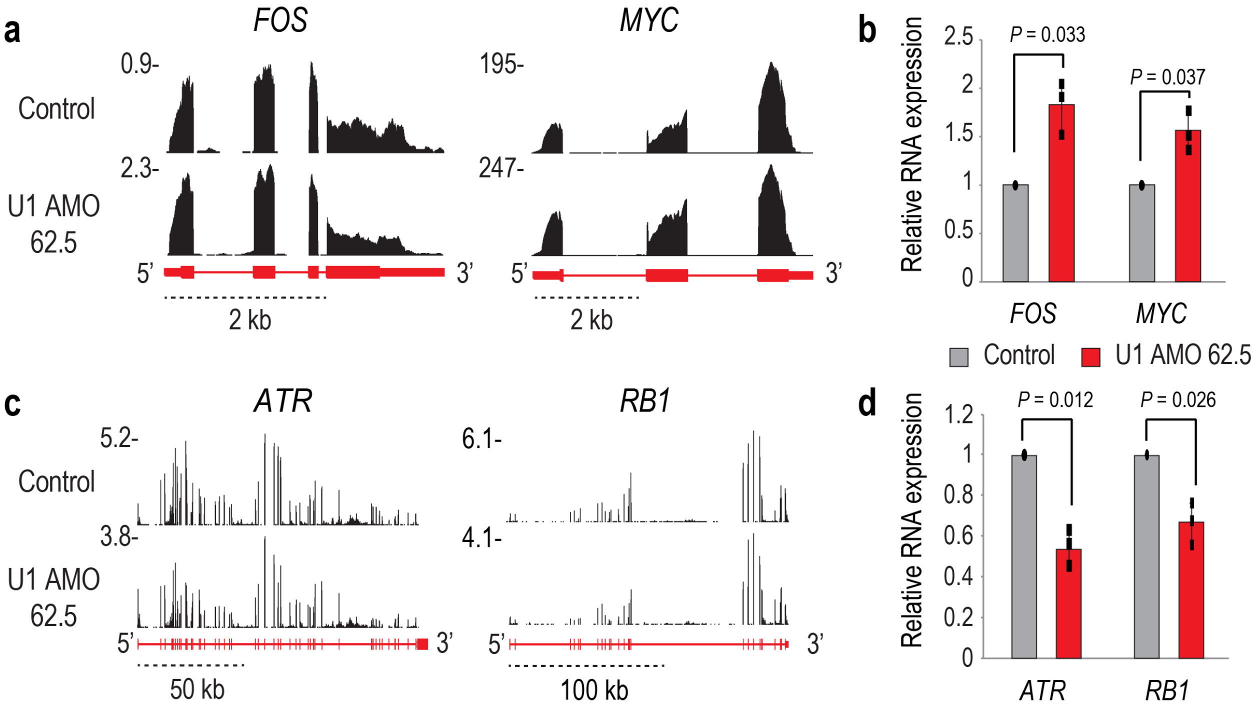

Supplementary Fig. 7. Validation of increased and decreased genes in low U1 AMO. Oncogenes and cancer related genes are increased and genes related to cell cycle and tumor suppressor function are decreased in the low U1 AMO. Genome browser view shows example of up-regulated genes, *FOS* and *MYC* (**a**), and down-regulated genes, *ATR* and *RB1* (**c**). Histogram shows up-regulated genes (**b**), and down-regulated genes (**d**) analyzed by RT-qPCR (mean ± SD; n=3, independent cell cultures). *P*-value was calculated with two-tailed Student's t-test. HeLa cells were incubated with 4-shU for last 2 hours after 6 hours of transfection with control or U1 AMO 62.5 pmole. 4-shU labeled RNA were extracted then ERCC RNA spike-in controls added to normalize the RT-qPCR data[3]. Source data are provided as a Source Data file.

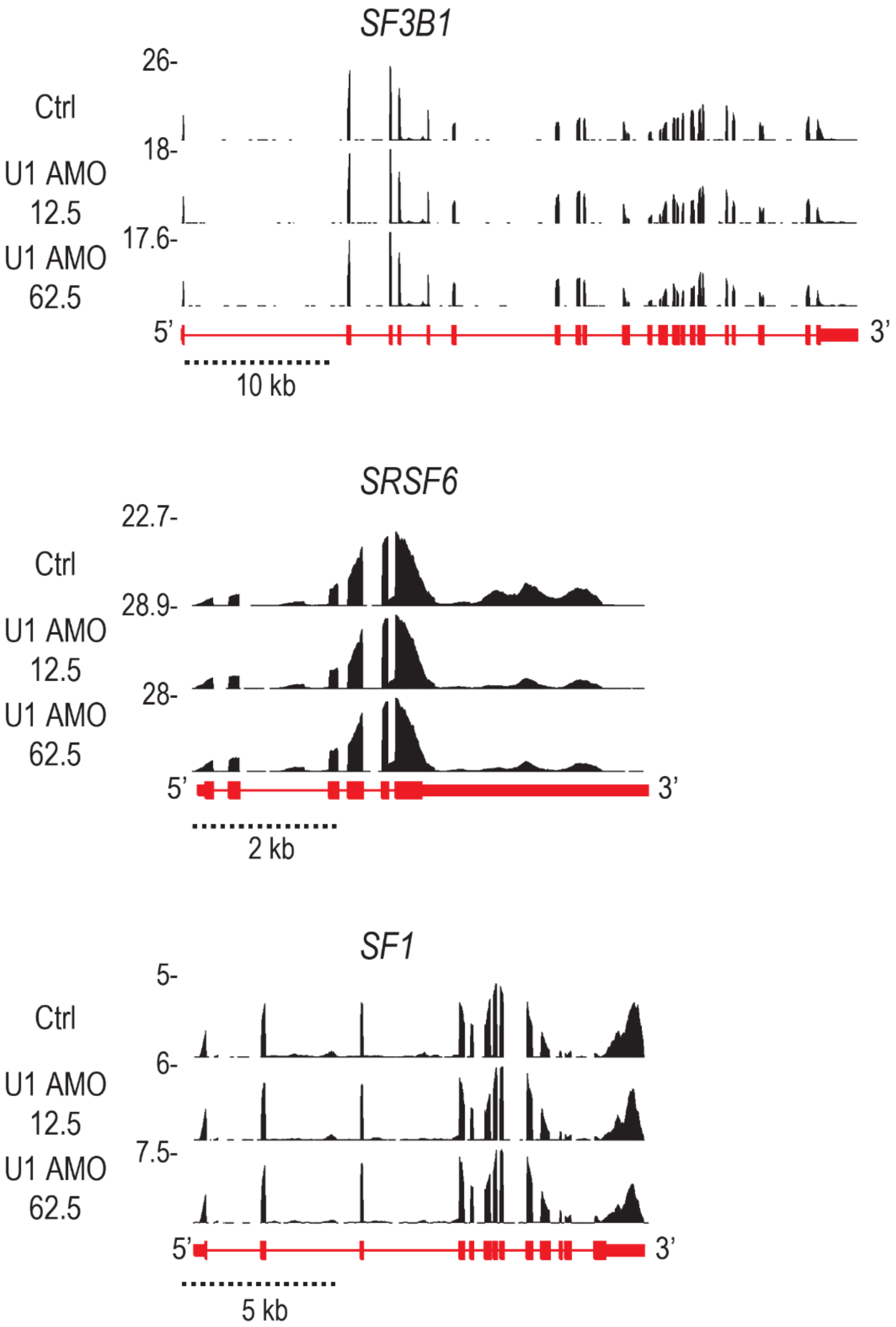

Supplementary Fig. 8. U1 AMO induces expression level changes in splicing factors. Genome browser view shows *SF3B1* decrease and *SRSF6* and *SF1* increases in both U1 AMO 12.5 and 62.5.

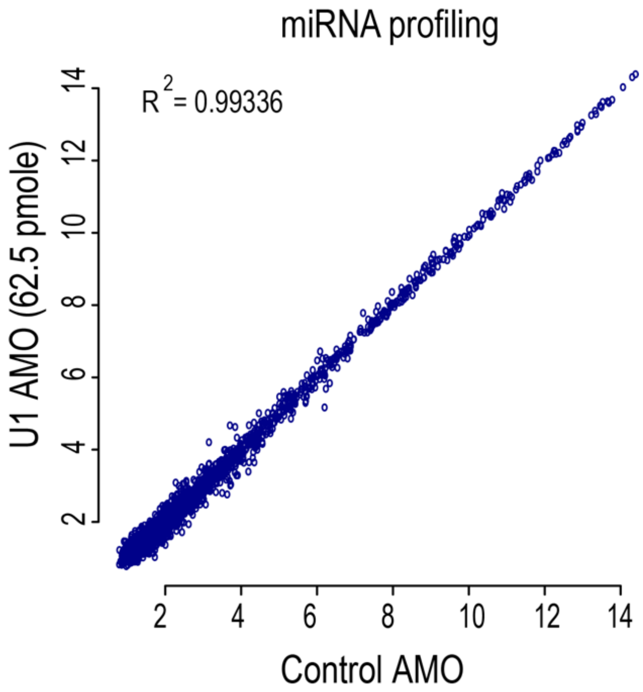

Supplementary Fig. 9. Moderate U1 decrease doesn't affect steady state miRNA level. A scatter plot of miRNAs expression levels in control cells versus cells transfected with moderate U1 AMO (62.5 pmole) for 8 hours. RNA extraction, microarray used, and analysis are as described in Methods.

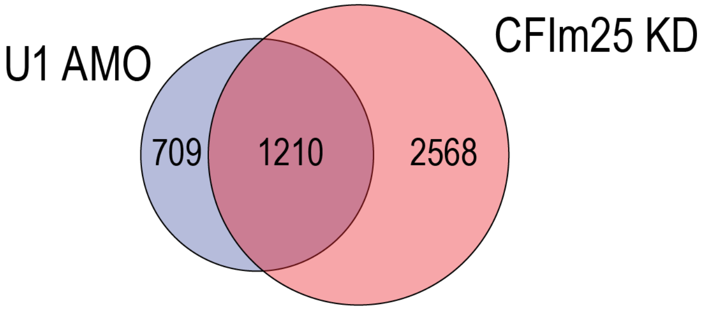

Supplementary Fig. 10. Both low U1 AMO and CFIm25/CPSF5 knockdown cause widespread 3'UTR shortening. Venn diagram of LECDS identified genes with 3'UTR shortening in both 12.5 and 62.5 pmole U1 AMO treatment (blue; 1,919 genes) versus CFIm25/CPSF5 knockdown (red; 3,778 genes) in HeLa cells.

Supplementary Tables

	U1 AMO (62.5 pmole)					U1 Over-expression						
	Migration index	Invasion index	Proliferation (% Increase)			Migration index		Invasion index		Proliferation (% Increase)		
	U1 AMO	U1 AMO	24hr	48hr	72hr	1μg	1.5μg	1μg	1.5μg	24hr	48hr	72hr
**HeLa** (cervical cancer)	500	463	0	38	38	74	52	67	37	No effect		
**A-549** (lung adenocarcinoma)	172	164	0	13	15	66	47	92	91	No effect		
**MCF-7** (breast adenocarcinoma)	158	n/a	0	10	12	n/a						
**MDA-MB-231** (breast adenocarcinoma)	170	153	0	5	7	57	54	63	72	No effect		

Supplementary Table 1. U1 level modulates migration and invasion in cancer cell lines. Migration and invasion changes with U1 AMO or U1 OE were determined as described in Fig 1 on HeLa, A-549, MCF-7, and MDA-MB-231 cells. Migration and invasion indexes are presented as percent of control for cells treated with U1 AMO (62.5 pmole) or U1 OE (both 1μg and 1.5μg) for 24 hours. Cell proliferation was assayed daily over three days.

	U1 AMO (pmole)			U1 Over-expression		
**Reads**	**Control AMO**	**12.5**	**62.5**	**Empty vector**	**1μg**	**1.5μg**
**Total**	64,965,053	57,555,134	60,562,764	226,028,904	185,947,498	207,313,315
**Mapped**	47,916,977	57,555,134	42,980,600	136,133,739	92,804,677	123,786,737
**% Mapped**	73.80%	71.30%	71.00%	60.20%	49.90%	59.70%

Supplementary Table 2. Summary of RNA-seq Data.

Total number of reads, mapped reads and the percentage of mapped reads for each RNA-seq sample are presented.

Transcriptome Changes		U1 AMO (pmole)			U1 Over-expression		
		**12.5**	**62.5**	overlap	**1µg**	**1.5µg**	overlap
**Differential expression**	Up-regulated	525	787	309 (59%)	1249	1594	1107 (89%)
	Down-regulated	969	1402	818 (84%)	2418	4430	2351 (97%)
**3'UTR length**	Total shortened	2530	2630	1919 (76%)	563	1063	344 (61%)
	Total lengthened	324	218	130 (60%)	4615	3831	3210 (84%)
**Alternative Splicing**	A5'SS	1542	1547	702 (45%)	1775	1653	817 (49%)
	A3'SS	1190	1163	499 (43%)	1508	1380	647 (47%)
	CE	721	705	247 (35%)	820	725	275 (38%)
	IR	334	483	152 (31%)	1859	1769	1059 (60%)
	MXE	6	7	3 (43%)	4	7	3 (43%)

Supplementary Table 3. Transcriptome changes induced by U1 level modulation with U1 AMO or U1 over-expression in HeLa cells compared to controls. Shown are the numbers of genes affected at each dose. mRNA up-regulated and down-regulated genes were determined using GFold[4] differential expression software with the following cutoffs: fold change >1.5 fold and p-value < 0.01. 3'UTR length changes were determined by calculating the ratio of RNA-seq reads in the last exon over the coding region in the last exon, abbreviated in the text as LECDS, in the indicated conditions versus their controls. Splicing changes were analyzed by JUM[5] with the following parameters: $|\Delta\Psi| \geq$ 10%, p-value < 0.05. Abbreviations for alternative splicing events: Alternative 5'/3' Splice Sites (A5'SS, A3'SS), Cassette Exon (CE), Intron Retention (IR), Mutually Exclusive Exons (MXE).

U1 AMO vs cancer gene	*CDC27, NOTCH1, RAD23B, GATA2, CCND3, RCC2, TCF3, NDRG1, TIMP2, AKT2, BCL6, TOP2A, SBDS, RAP1GDS1, JAK1, CDKN2D, CDC34, VAV2, CCNB1, ARNT, EPS8, MET, MYH9, NUP98, TGFBI, ASXL1, MXI1, CREBBP, MITF, JAZF1, ARHGEF12, TRIM33, EWSR1, EIF4A2, ATRX, CDK6, TFG, TOP1, SKP2, ARID1A, EML4, SF3B1, CLTC, REL, CTNNA1, CCDC6, CDK4, GNAQ, TOP2B, ELK4, IGF1R, MYC, YES1, NOTCH2, PHB, CDK2, CBLB, TAP1, BTG1, CD44, GTF2H1, CCND1, HOXC13, TNFAIP3, NPM1, CBFB, RUNX1, FSTL3, KIAA1549, AXL, LIFR, RREB1, NFIB, NF1, ELK3, ECT2, TNFAIP1, PBRM1, GOPC, NFE2L2, CCNT1, ETS2, EP300, DDX5, CDC25A, TIMP3, TFRC, WHSC1, DICER1, VHL, PICALM, GLI3, MN1, PRKAR1A, MSN, RB1, ACSL3, FBXW7, SRI, EGFR, CKS2, WNT5A, CDK12, EPS15, ZNF331, HEY1, H3F3A, NSD1, FUS, ATF1, BMI1, MDM2, WWTR1, NOV, IL7R, SFPQ, NIN, LPP*
U1 OE vs cancer gene	*MXI1, CREBBP, MITF, JAZF1, ARHGEF12, TRIM33, EWSR1, EIF4A2, ATRX, CDK6, TFG, TOP1, SKP2, ARID1A, EML4, SF3B1, CLTC, REL, CTNNA1, CCDC6, CDK4, GNAQ, TOP2B, ELK4, IGF1R, MYC, YES1, NOTCH2, MUTYH, MLH1, TPM4, RALA, BCL2L2, CD74, TGFBR3, CANT1, PCNA, RBL2, EZH2, RPN1, FANCA, SDC4, BCOR, BRIP1, BMPR1A, CDC25B, BLM, HFE, LRIG3, HERPUD1, TGFBR2, TRIP11, TET2, BAG1, NCOA2, MNAT1, LYN, GNA11, FOXP1, PBX1, GMPS, BIRC3, ABL2, PCSK7, TPR, CDC25C, PIM1, PALB2, ETV5, MKL1, SKI, SMARCA4, IGFBP7, FOS, CTGF, CREB1, BRCA1, RASA1, CDKN2C, AKAP9, BCL7A, NONO, CCNH, BCL2, EZR, SDHB, TGFBR1, MAFG, BRAF, E2F1, FGFR1, WHSC1L1, FYN, RAP2A, PCM1, APC, GLI2, HOXC11, ERBB3, BUB1B, RPL22, BUB1, PER1, CRK, RAD52, PRDM1, ZNF35, NR4A3, SUZ12, NUP214, VTI1A, MALT1, CARS, EXT2, PHB, CDK2, CBLB, TAP1, BTG1, CD44, GTF2H1, CCND1, HOXC13, TNFAIP3, NPM1, CBFB, RUNX1, FSTL3, KIAA1549, AXL, LIFR, RREB1, NFIB, NF1, ELK3, ECT2, TNFAIP1, PBRM1, GOPC, NFE2L2, CCNT1, ETS2, EP300, DDX5, CDC25A, TIMP3, TFRC, WHSC1, DICER1, VHL, PICALM, GLI3, MN1, PRKAR1A, MSN, RB1, ACSL3, FBXW7, SRI, EGFR, CKS2, WNT5A, CDK12, EPS15, ZNF331, HEY1, H3F3A, NSD1, FUS, ATF1, BMI1, MDM2, WWTR1, NOV, IL7R, SFPQ, NIN, LPP*

Supplementary Table 4. Cancer genes affected by U1 AMO and U1 over-expression.

Table showed the overlapped genes between cancer genes and 3’UTR length changed genes in U1 AMO and U1 OE samples.

FOS F	TCTCCAGTGCCAACTTCATT
FOS R	GCTCTGGTCTGCGATGG
MYC F	CATACATCCTGTCCGTCCAAG
MYC R	GAGTTCCGTAGCTGTTCAAGT
ATR F	GGTACCCAGAATTGATGGAAGT
ATR R	GCTGGTAGAAGAAGCAACATTTAG
CDC42 3'RACE 1st F	ACTCACCACTGTCCAAAGACTCCT
CDC42 3'RACE 2nd F	ACCTGAAGGCTGTCAAGTATGTGG
RB1 F	CAATCAAAGGACCGAGAAGGA
RB1 R	GTGTGATTATTCTGGAGAGGAAGA
CDC25A 3'RACE 1st F	AAG AGC AAG AGG GAG ATG TAC AG
CDC25A 3'RACE 2nd F	GGA GCC CAG GGC ATC TTG CTG GC
TRAF4 3'RACE 1st F	TCTGGAAGATTGGCAGCTATG
TRAF4 3'RACE 2nd F	CCCAGCCTTCTACACACATAAG
MAP2K1 3'RACE 1st F	TGG CTC TGC TCC ACC ATC GGC CT
MAP2K1 3'RACE 2nd F	GTG CTT GGG GCT ATT TGT GTG TA
PRKAA2 exon8-9 F	CTGGTTGATAACAGGAGCTATCTT
PRKAA2 exon8-9 R	CAGGCGAGGTGAAACTGAA
PRKAA2 new exon8-9 F	ACCTCTTACCTGGCATCTTTC
PRKAA2 new exon8-9 R	CAATGTGCTTCCGGTCAAAG
MRPL18 new exon1-2 F	CGAAGACCACAGTAGGAAGTTAAG
MRPL18 new exon1-2 R	AGAACTCACGGGAGGGAAA
KIAA1524 exon1-2 F	TTGAAGTCCTTGCTCCTGAC
KIAA1524 exon1-2 R	GTTGAGACAGCAAACCGATAATAC
GAPDH F	ACCACAGTCCATGCCATCAC
GAPDH R	TCCACCACCCTGTTGCTGTA

Supplementary Table 5. Primers used for RT-qPCR, 3'RACE and RT-PCR

(Supplementary Figures 5-7)

Supplementary references

1. Berg, M.G. et al. U1 snRNP determines mRNA length and regulates isoform expression. *Cell* **150**, 53-64 (2012).
2. Masamha, C.P. et al. CFIm25 links alternative polyadenylation to glioblastoma tumour suppression. *Nature* **510**, 412-416 (2014).
3. Oh, J.M. et al. U1 snRNP telescripting regulates a size-function-stratified human genome. *Nat Struct Mol Biol* **24**, 993-999 (2017).
4. Feng, J. et al. GFOLD: a generalized fold change for ranking differentially expressed genes from RNA-seq data. *Bioinformatics* **28**, 2782-2788 (2012).
5. Wang, Q. & Rio, D.C. JUM is a computational method for comprehensive annotation-free analysis of alternative pre-mRNA splicing patterns. *Proc Natl Acad Sci U S A* **115**, E8181-E8190 (2018).
