# Supplementary material for: U1 snRNP regulates cancer cell migration and invasion in vitro
Source: Nat Commun. 2020 Jan 7;11:1. doi: 10.1038/s41467-019-13993-7 (PMC6946686; doi:10.1038/s41467-019-13993-7)
Supplement: Supplementary file 2 — Reporting Summary [file 41467_2019_13993_MOESM2_ESM.pdf]

nature research

Corresponding author(s): Gideon Dreyfuss

Last updated by author(s): Nov 12, 2019

Reporting Summary

Nature Research wishes to improve the reproducibility of the work that we publish. This form provides structure for consistency and transparency in reporting. For further information on Nature Research policies, see Authors & Referees and the Editorial Policy Checklist.

Statistics

For all statistical analyses, confirm that the following items are present in the figure legend, table legend, main text, or Methods section.

n/a	Confirmed	
☐	☒	The exact sample size (*n*) for each experimental group/condition, given as a discrete number and unit of measurement
☐	☒	A statement on whether measurements were taken from distinct samples or whether the same sample was measured repeatedly
☐	☒	The statistical test(s) used AND whether they are one- or two-sided<br>*Only common tests should be described solely by name; describe more complex techniques in the Methods section.*
☐	☒	A description of all covariates tested
☐	☒	A description of any assumptions or corrections, such as tests of normality and adjustment for multiple comparisons
☐	☒	A full description of the statistical parameters including central tendency (e.g. means) or other basic estimates (e.g. regression coefficient) AND variation (e.g. standard deviation) or associated estimates of uncertainty (e.g. confidence intervals)
☐	☒	For null hypothesis testing, the test statistic (e.g. *F*, *t*, *r*) with confidence intervals, effect sizes, degrees of freedom and *P* value noted<br>*Give P values as exact values whenever suitable.*
☒	☐	For Bayesian analysis, information on the choice of priors and Markov chain Monte Carlo settings
☐	☒	For hierarchical and complex designs, identification of the appropriate level for tests and full reporting of outcomes
☐	☒	Estimates of effect sizes (e.g. Cohen's *d*, Pearson's *r*), indicating how they were calculated

Our web collection on statistics for biologists contains articles on many of the points above.

Software and code

Policy information about availability of computer code

Data collection	UCSC (http://genome.ucsc.edu) genome data was used for reference genomes and miRNA regulatory sites.
Data analysis	STAR v.2.6.0b, JUM v.2.0.2

For manuscripts utilizing custom algorithms or software that are central to the research but not yet described in published literature, software must be made available to editors/reviewers. We strongly encourage code deposition in a community repository (e.g. GitHub). See the Nature Research guidelines for submitting code & software for further information.

Data

Policy information about availability of data

All manuscripts must include a data availability statement. This statement should provide the following information, where applicable:

- Accession codes, unique identifiers, or web links for publicly available datasets
- A list of figures that have associated raw data
- A description of any restrictions on data availability

All sequencing data described are available on GEO under the accession number (GSE140543).

Field-specific reporting

Please select the one below that is the best fit for your research. If you are not sure, read the appropriate sections before making your selection.

☒ Life sciences ☐ Behavioural & social sciences ☐ Ecological, evolutionary & environmental sciences

For a reference copy of the document with all sections, see nature.com/documents/nr-reporting-summary-flat.pdf

Life sciences study design

All studies must disclose on these points even when the disclosure is negative.

Sample size	To run a two-tailed Student's t-test, we needed at least 3 repeats for each experiment.
Data exclusions	No data were excluded from the analyses.
Replication	We repeated at least 3 times for real-time PCR, cell proliferation, migration and invasion assay.
Randomization	We randomly select cells for each control or U1 AMO or U1 OE transfection.
Blinding	We were not blinded to the experimental groups during the analysis,however data assessment was conducted in a blinded fashion.

Reporting for specific materials, systems and methods

We require information from authors about some types of materials, experimental systems and methods used in many studies. Here, indicate whether each material, system or method listed is relevant to your study. If you are not sure if a list item applies to your research, read the appropriate section before selecting a response.

Materials & experimental systems

n/a	Involved in the study	
☒	☐	Antibodies
☐	☒	Eukaryotic cell lines
☒	☐	Palaeontology
☒	☐	Animals and other organisms
☒	☐	Human research participants
☒	☐	Clinical data

Methods

n/a	Involved in the study	
☐	☒	ChIP-seq
☒	☐	Flow cytometry
☒	☐	MRI-based neuroimaging

Eukaryotic cell lines

Policy information about cell lines

Cell line source(s)	We used HeLa, A549, MCF-7 and MDA-MB-231 cells obtained from ATCC.
Authentication	None of the cell lines used were authenticated.
Mycoplasma contamination	All cell lines were tested for mycoplasma contamination.
Commonly misidentified lines (See ICLAC register)	N/A

ChIP-seq

Data deposition

☒ Confirm that both raw and final processed data have been deposited in a public database such as GEO.

☐ Confirm that you have deposited or provided access to graph files (e.g. BED files) for the called peaks.

Data access links
May remain private before publication.

https://www.ncbi.nlm.nih.gov/geo/query/acc.cgi?acc=GSE140543

Files in database submission

Oh_NCOMMS-19-25148A-Z_metadata_info.xls
cAMO_for_U1AMO_1.fastq.gz
cAMO_for_U1AMO_2.fastq.gz
U1AMO_12.5pmol_1.fastq.gz
U1AMO_12.5pmol_2.fastq.gz
U1AMO_62.5pmol_1.fastq.gz
U1AMO_62.5pmol_2.fastq.gz
hele_overexpression_control_1.fastq.gz
hele_overexpression_control_2.fastq.gz
hele_overexpression_1ug_1.fastq.gz
hele_overexpression_1ug_2.fastq.gz
hele_overexpression_1.5ug_1.fastq.gz

hele_overexpression_1.5ug_2.fastq.gz
cAMO_for_U1AMO.norm.bw
U1AMO_12.5pmol.norm.bw
U1AMO_62.5pmol.norm.bw
hele_overexpression_control.norm.bw
hele_overexpression_1ug.norm.bw
hele_overexpression_1.5ug.norm.bw

Genome browser session
(e.g. UCSC)

http://genome.ucsc.edu/cgi-bin/hgTracks?db=hg38&lastVirtModeType=default&lastVirtModeExtraState=&virtModeType=default&virtMode=0&nonVirtPosition=&position=chr8%3A127736069%2D127741434&hgsid=778256413_rJ4AaIOR9e3HaIGPdafCAOeFr5H3

Methodology

Replicates

We report six datasets. Two for U1 AMO (12.5 and 62.5 picomoles), two for U1 over-expression (OE) (1µg and 1.5µg), and two controls, for the AMO and OE, respectively, as detailed in the Methods. Each sample was first compared to the corresponding control. As shown in Figure 1, the biological effects (oncogenicity-related phenotypic changes) observed at the two U1 AMO doses were nearly the same (within 20%), and the deep RNA-seq detected highly similar changes in both, they represent replicates. Importantly, all the conclusions described in the manuscript are based only on changes detected at both U1 AMO and U1 OE, respectively. The same applies to the U1 OE. Additional experiments and direct data validation, include genome browser images of multiple genes, and 3'RACE, are described in the manuscript.

Sequencing depth

Control AMO total mapped reads : 64,965,053, Mapped reads: 47,916,977, % of mapped reads : 73.8 %
12.5 U1 AMO total mapped reads : 57,555,134, Mapped reads: 57,555,134, % of mapped reads : 71.30%
62.5 U1 AMO total mapped reads : 60,562,764, Mapped reads: 42,980,600, % of mapped reads : 71.00%
Control empty vector total mapped reads : 226,028,904, Mapped reads: 136,133,739, % of mapped reads : 60.20%
1ug U1 OE total mapped reads : 185,947,498, Mapped reads: 92,804,677, % of mapped reads : 49.90%
1.5ug U1 OE total mapped reads : 207,313,315, Mapped reads: 123,786,737, % of mapped reads : 59.70%
We used 125bp paired-end reads.

Antibodies

N/A

Peak calling parameters

N/A

Data quality

N/A

Software

Illumina Casava1.9 software used for basecalling. Reads were trimmed of adapter sequences using TrimGalore, and then were aligned to the reference genome (UCSC, hg38) using STAR by default parameters. Reads with multiple alignments were filtered for downstream analysis. FPKMs for RNA-seq were generated using GFold with default parameters. 3'UTR shortening was calculated using the new LECDS method where 3'UTR signals were compared to that from the last exon's coding sequence. The significance of this read change was detected using a Fisher's Exact test followed by Benjamini–Hochberg multiple testing with an adjusted P-value ≤ 0.01
